# Device Selection for Transcatheter Aortic Valve Implantation

**DOI:** 10.3390/jcm12010284

**Published:** 2022-12-29

**Authors:** Camille Granger, Paul Guedeney

**Affiliations:** ACTION Study Group, INSERM UMRS 1166 Institut de Cardiologie, Sorbonne Université, Pitié Salpêtrière (APHP), 75013 Paris, France

Twenty years after the first implantation by Alain Cribier and his team, transcatheter aortic valve implantation (TAVI) has demonstrated its efficacy and safety in patients with symptomatic severe aortic stenosis with high, intermediate, and even low surgical risk. Consequently, its indications have expanded widely over the years, with the improvement of structural devices, operator’s experience, procedural techniques, and post-procedural management [1,2]. 

Most devices currently available are either balloon-expandable or self-expanding transcatheter heart valves (THV). A recent meta-analysis of randomized control trials and a prospective international observational study suggested that newer generation devices such as Sapien 3 for the Edward Sapien™ (Edward Lifesciences, Irvine, CA, USA) or the Evolut R or Evolut Pro for the Medtronik CoreValve™ (Minneapolis, MN, USA) were associated with a reduction of all-cause mortality, and major cerebro-cardiovascular events when compared to surgical aortic valve replacement (SAVR) [3,4]. There are, however, only limited conclusive data on the respective performance of these two device types. In fact, both types were recently compared, after propensity score matching, among 12,381 patients undergoing transfemoral TAVI and included in the international Cerebrovascular Events in Patients Undergoing Transcatheter Aortic Valve Implantation (CENTER) collaboration [5]. No significant differences were reported for “hard” clinical endpoints, such as death or stroke—neither at thirty days, nor one year. 

Compared to SAVR, TAVI has been traditionally associated with a higher risk of aortic regurgitation due to paravalvular leakage or permanent pacemaker implantation (PPI) [3,4]. The presence of moderate or severe paravalvular leak following TAVI remains a source of concern as it was an independent risk factor for mortality in the French national FRANCE TAVI registry, with six and a half years of follow-up [6]. 

The recent long-term study by Tamm et al. reported higher mean transaortic gradients but lower paravalvular leak with Sapien 3 prosthesis when compared to the Evolut R, although this did not result in any significant difference concerning five-year mortality, which was similar between the two types of devices [7]. The recent Evolut vs. Myval for the Treatment of Patients with Severe Aortic Valve Stenosis (EVAL) registry reported promising results on the safety and efficacy of the new Myval balloon-expandable THV, which provided comparable performance to the Evolut R self-expanding device, with a lower rate of PPI and a moderate to severe paravalvular leak six months after the procedure [8]. The self-expanding Acurate neo™ (Boston scientific, Malborough, Massachusetts, USA) and Portico™ (Abbott, Chicago, IL, USA) THV were compared in a large multicenter center observational study with propensity-score matching, which did not report significant differences in terms of grade II or more paravalvular leakage (4.8% vs. 3.5%, respectively, *p* = 0.546) [9].

Device selection remains a challenge in patients with small aortic annuli, as it increases the risk of prosthesis-patient mismatch (PPM) which is associated with poorer outcomes, or in cases of severely calcified aortic valves which can result in a higher risk of paravalvular leakage, PPI or even aortic annulus rupture. Balloon-expandable Sapien 3 valves have been associated with significantly higher rates of PPM in comparison with self-expanding Evolut Pro or Accurate Neo valves, while mild to severe paravalvular leaks may be more frequent with the latter [10]. Similar results were reported in an echo Doppler comparative study in patients with small aortic annuli, demonstrating improved hemodynamic performances with self-expanding devices when compared to balloon-expandable valves [11]. It should be noted, however, that these features did not result in differences in terms of clinical outcomes, although the relatively limited sample size and follow-up duration prevent any definitive conclusions. In the case of patients with severely calcified aortic valve stenosis, self-expanding new-generation Evolut Pro devices have been associated, yet again, with lower rates of moderate and severe PPM compared to balloon-expandable Sapien 3, without significant differences in terms of paravalvular leakage or PPI implantation [12].

Uncertainty remains about the selection of the optimal device in patients with severe aortic stenosis undergoing TAVR. Larger observational studies and randomized controlled trials with adequate statistical power and long-term follow-up are warranted to further evaluate this issue.
